# Harnessing artificial intelligence in sepsis care: advances in early detection, personalized treatment, and real-time monitoring

**DOI:** 10.3389/fmed.2024.1510792

**Published:** 2025-01-06

**Authors:** Fang Li, Shengguo Wang, Zhi Gao, Maofeng Qing, Shan Pan, Yingying Liu, Chengchen Hu

**Affiliations:** ^1^Department of General Surgery, Chongqing General Hospital, Chongqing, China; ^2^Department of Stomatology, The Second Affiliated Hospital of Chongqing Medical University, Chongqing, China

**Keywords:** artificial intelligence, sepsis management, early detection, personalized treatment, real-time monitoring

## Abstract

Sepsis remains a leading cause of morbidity and mortality worldwide due to its rapid progression and heterogeneous nature. This review explores the potential of Artificial Intelligence (AI) to transform sepsis management, from early detection to personalized treatment and real-time monitoring. AI, particularly through machine learning (ML) techniques such as random forest models and deep learning algorithms, has shown promise in analyzing electronic health record (EHR) data to identify patterns that enable early sepsis detection. For instance, random forest models have demonstrated high accuracy in predicting sepsis onset in intensive care unit (ICU) patients, while deep learning approaches have been applied to recognize complications such as sepsis-associated acute respiratory distress syndrome (ARDS). Personalized treatment plans developed through AI algorithms predict patient-specific responses to therapies, optimizing therapeutic efficacy and minimizing adverse effects. AI-driven continuous monitoring systems, including wearable devices, provide real-time predictions of sepsis-related complications, enabling timely interventions. Beyond these advancements, AI enhances diagnostic accuracy, predicts long-term outcomes, and supports dynamic risk assessment in clinical settings. However, ethical challenges, including data privacy concerns and algorithmic biases, must be addressed to ensure fair and effective implementation. The significance of this review lies in addressing the current limitations in sepsis management and highlighting how AI can overcome these hurdles. By leveraging AI, healthcare providers can significantly enhance diagnostic accuracy, optimize treatment protocols, and improve overall patient outcomes. Future research should focus on refining AI algorithms with diverse datasets, integrating emerging technologies, and fostering interdisciplinary collaboration to address these challenges and realize AI’s transformative potential in sepsis care.

## 1 Introduction

Sepsis, a life-threatening condition caused by the body’s extreme immune response to infection, is a leading cause of global morbidity and mortality. Characterized by dysregulated immune responses, it triggers widespread inflammation, tissue damage, and organ failure (1). The rapid and often fatal progression of sepsis underscores the urgent need for innovative approaches to improve early detection and management. However, despite advances in medical research, sepsis remains challenging to diagnose and treat due to its heterogeneous nature and variable progression (2). Recent advancements in Artificial Intelligence (AI) offer transformative potential to address these challenges, promising significant improvements in diagnosis, treatment, and overall patient outcomes (3).

AI, leveraging machine learning (ML) and deep learning techniques, has demonstrated substantial success in healthcare by processing large, complex datasets to identify patterns often missed by traditional methods (4). In sepsis management, AI applications have been developed to enhance early detection, predict disease progression, and personalize treatment strategies (5). For instance, Baghela et al. (6) utilized AI to analyze gene expression signatures, enabling earlier and more precise triage of sepsis patients in 2022. Similarly, Wang et al. (7) developed a machine learning model using EHR data to predict sepsis onset, achieving high accuracy with an AUC of 0.91 in 2021. These studies demonstrate AI’s potential to accelerate diagnosis and facilitate timely interventions (8).

Despite these advancements, the integration of AI into sepsis care faces significant challenges. A major limitation is the variability in model performance across different institutions and patient populations. AI models trained on datasets from specific populations often struggle to generalize to other settings due to demographic and geographic variability (9). For example, sepsis prediction models developed in high-resource healthcare environments may underperform in resource-limited settings, where clinical workflows and patient profiles differ markedly. Addressing this limitation requires the inclusion of diverse, representative datasets during model development to improve robustness and applicability (10).

Another limitation is the lack of universal applicability of AI models in dynamic clinical environments. Many AI algorithms rely on static data, which may not adequately capture the rapidly changing conditions of sepsis patients. Developing real-time, adaptive AI systems capable of continuously learning from new data is critical to ensuring broader clinical utility (10). Moreover, the complexity of AI models can create a disconnect between their predictive capabilities and their practical implementation. Clinicians often find it challenging to interpret AI-generated outputs, which can hinder trust and adoption in critical care settings (7).

AI’s role extends beyond detection to include prognosis and personalized treatment strategies. For instance, Fan et al. (8) developed ML models to predict outcomes for patients with sepsis-associated acute kidney injury (S-AKI), enhancing the accuracy of mortality predictions and guiding clinical interventions in 2023. Similarly, Petersen et al. (11) used reinforcement learning to simulate adaptive treatment strategies, demonstrating improved outcomes in simulated patients in 2019. These advancements highlight the potential for AI to provide tailored, data-driven treatment plans that address the variability of sepsis presentations and patient responses (6).

**Figure 1:**
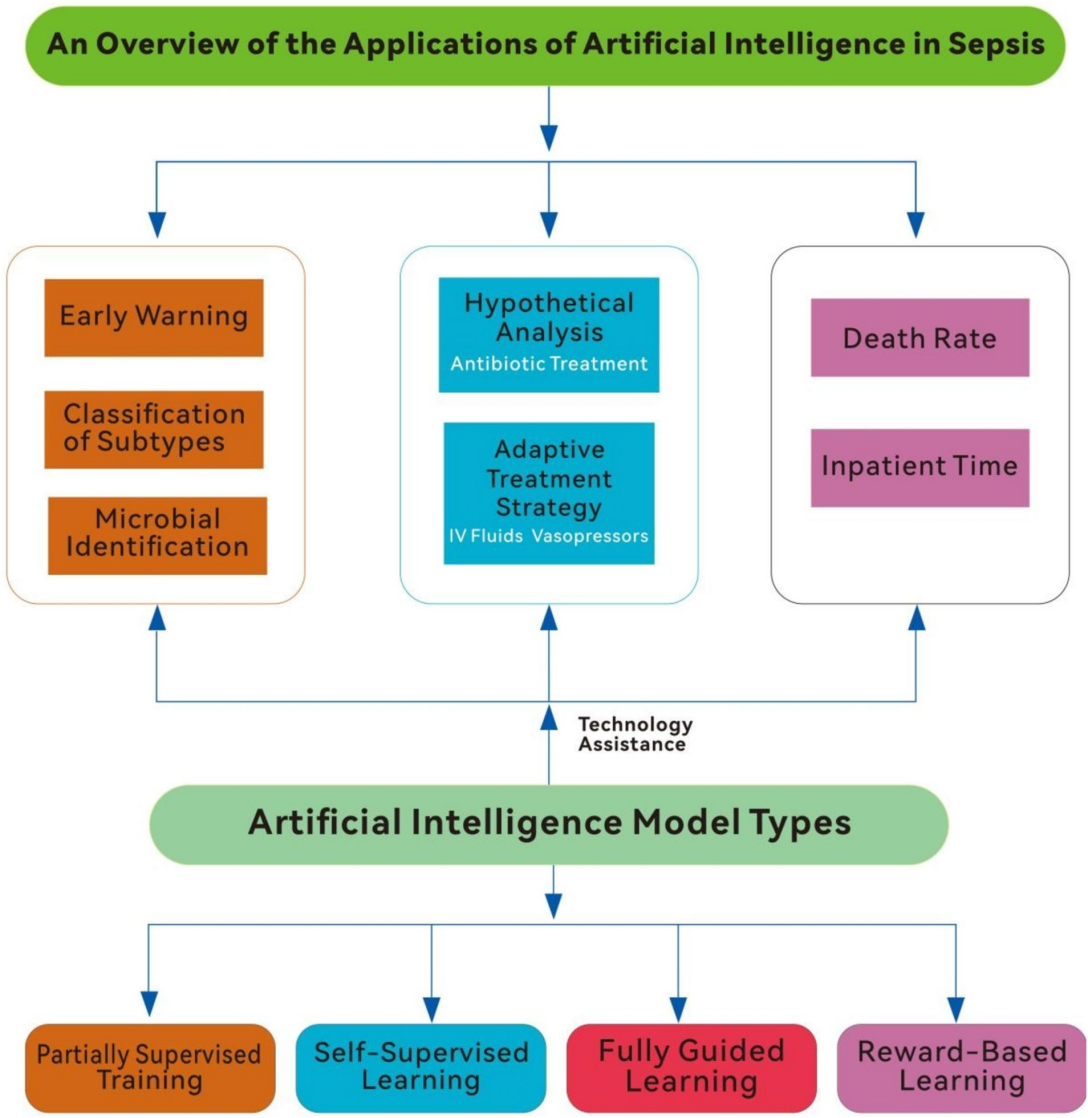
An overview of the application of artificial intelligence in sepsis (IV fluids, intravenous fluids).

In conclusion, AI offers a promising avenue for revolutionizing sepsis care, from early detection to personalized treatment. However, its clinical adoption requires addressing significant challenges, including the need for generalizability, adaptability, and better integration into clinical workflows. By tackling these limitations and harnessing AI’s predictive power, healthcare providers can enhance diagnostic accuracy, optimize treatments, and improve patient outcomes. This review explores recent advancements in AI applications for sepsis management, emphasizing its contributions, challenges, and future directions (Figure 1).

## 2 AI in early detection and prediction of sepsis

Early detection and prediction of sepsis are essential for improving patient outcomes, as timely interventions can substantially reduce morbidity and mortality. Traditional sepsis diagnosis methods rely on clinical assessments and laboratory tests, which may not detect the condition in its early stages due to nonspecific symptoms. Recently, AI has become a powerful tool for the early detection and prediction of sepsis by analyzing vast patient data to identify early warning signs and enhance clinical decision-making (11). AI techniques, such as machine learning (ML) and deep learning, are transforming sepsis detection and management. ML models are particularly effective when applied to electronic health records (EHRs) to predict sepsis onset (8).

One promising application of AI in sepsis is the development of ML models to predict sepsis onset using EHRs. Wang et al. (7) developed a random forest algorithm that achieved an AUC (Area under the Receiver Operating Characteristic Curve) of 0.91, demonstrating high accuracy in analyzing clinical variables from EHR data. Similarly, these studies emphasize the significant potential of AI in enhancing early detection and facilitating timely interventions (12).

AI models have also advanced through the integration of structured and unstructured clinical data for early sepsis prediction. Goh et al. (13) introduced the SERA algorithm, which combines structured data with unstructured clinical notes to predict sepsis. The algorithm achieved an AUC of 0.94, with sensitivity and specificity of 0.87, outperforming traditional methods by predicting sepsis up to 12 h before clinical onset. This highlights the importance of diverse data integration for improving accuracy and timeliness. Giacobbe et al. (14) further emphasized AI’s potential in early sepsis detection, proposing that a multidisciplinary approach to model development could enhance clinical utility and decision-making.

To provide a comprehensive overview of the latest AI-driven approaches to sepsis detection, prediction, and treatment, key studies are summarized in Table 1: Comparative Summary of AI Models in Sepsis Management. This table highlights the performance of various machine learning (ML) models, neural networks, and deep learning techniques, including their Area under the Receiver Operating Characteristic Curve (AUC), sensitivity, and specificity metrics. By detailing these performance indicators, the table offers valuable insights into the strengths and limitations of AI methods across different stages of sepsis care, emphasizing their clinical potential and challenges.

**Table 1:** Comparative summary of AI models in sepsis management.

Title	Reference	Model	Application	AUC	Sensitivity	Specificity
Early detection of sepsis utilizing deep learning on electronic health record event sequences	Lauritsen et al. (44)	Deep learning	Early warning of sepsis	0.85	0.88	0.76
A novel artificial intelligence based intensive care unit monitoring system: using physiological waveforms	Mollura et al. (17)	Machine learning	Early warning of sepsis	0.81	0.85	0.78
Prospective, multi-site study of patient outcomes after implementation of the TREWS early warning system	Adams et al. (119)	Machine learning	Early warning of sepsis	0.87	0.89	0.84
Predicting sepsis onset using a machine learned causal probabilistic network algorithm based on EHR data	Valik et al. (120)	Machine learning	Early warning of sepsis	0.92	0.85	0.81
Machine learning of cell population data for early prediction of bacteremia among adult patients	Chang et al. (121)	Machine learning	Early warning of sepsis	0.88	0.90	0.84
Integrated biosensor for rapid and point-of-care sepsis diagnosis	Min et al. (122)	Biosensor (IL-3)	Early detection of sepsis	0.91	0.92	0.89
Contextual embedding’s from clinical notes improves prediction of sepsis	Amrollahi et al. (123)	Clinical Text Embed-dings	Early prediction of sepsis	0.86	0.88	0.84
Early predicting 30-day mortality in sepsis in MIMIC-III by an artificial neural networks model	Su et al. (124)	Artificial Neural Networks	Early prediction of mortality in sepsis	0.89	0.91	0.87
Prediction of sepsis in the intensive care unit with minimal electronic health record data	Desautels et al. (39)	Machine Learning	Sepsis prediction with minimal EHR data	0.88	0.86	0.83
Unsupervised learning approach for predicting sepsis onset in ICU patients	Ramos et al. (125)	Unsuper-vised learning	Sepsis onset prediction	0.84	0.86	0.82
Predicting the onset of sepsis using vital signs data: a machine learning approach	Tran et al. (126)	Machine Learning	Early detection of sepsis using vital signs	0.88	0.87	0.85
The application of artificial intelligence in the management of sepsis	Yang et al. (46)	Machine Learning	Prediction, early detection, and treatment	0.89	0.91	0.88
A time-phased machine learning model for real-time prediction of sepsis in critical care	Li et al. (127)	Machine Learning	Real-time prediction of sepsis	0.91	0.92	0.90
Derivation, validation, and potential treatment implications of novel clinical phenotypes for sepsis	Seymour et al. (128)	Machine learning	Subtyping analysis of sepsis	0.89	0.88	0.85
Utilization of deep learning for subphenotype identification in sepsis-associated acute kidney injury	Chaudhary et al. (129)	Deep learning	Subtyping analysis of sepsis	0.87	0.89	0.86
The AI clinician learns optimal treatment strategies for sepsis in intensive care	Komorowski et al. (40)	Machine learning	Precision treatment of sepsis	0.89	0.88	0.84
Prediction of sepsis and in-hospital mortality using electronic health records	Khojandi et al. (130)	Machine Learning	Mortality prediction	0.86	0.87	0.85

AI applications in pediatric sepsis address unique challenges posed by nonspecific symptoms. Le et al. (15) developed an ML algorithm achieving an AUC of 0.916 for detecting severe sepsis at onset and 0.718 for predicting sepsis 4 h prior, outperforming traditional scoring systems like the Pediatric Logistic Organ Dysfunction (PELOD-2) and Systemic Inflammatory Response Syndrome (SIRS) scores. Similarly, Honoré et al. (11) developed a model using vital signs to predict neonatal sepsis, achieving an AUC of 0.82, underscoring the value of non-invasive monitoring in neonatal care.

AI-based models also hold promise in ED settings. Kijpaisalratana et al. (16) compared ML models to traditional screening tools like qSOFA (Quick Sequential Organ Failure Assessment), MEWS (Modified Early Warning Score), and SIRS for early sepsis detection in emergency patients. The ML models outperformed traditional methods, with the random forest algorithm achieving an AUC of 0.931.This study underscores AI’s potential to enhance sepsis screening and early detection in emergency settings, where rapid diagnosis is crucial for patient outcomes (17). Liu et al. (18) further highlighted the superior performance of ML-based predictive models over classical approaches like SOFA and qSOFA in patients with acute pancreatitis.

Building on these advancements in early detection, AI also plays a crucial role in improving diagnostic accuracy and efficiency. Leveraging ML and data analytics, AI significantly improves diagnostic processes, supporting clinicians in making more informed and precise decisions. Machine learning models, such as random forests and support vector machines, are widely used for early sepsis detection due to their ability to handle structured datasets from electronic health records (EHRs). Random forests, for instance, are effective in identifying early warning signs of sepsis by analyzing a combination of clinical variables, such as vital signs and laboratory results. These models excel in ICU settings, where large volumes of structured data are available. Moreover, their interpretability allows clinicians to understand how specific variables contribute to predictions, fostering trust in their use.

In resource-limited environments, simpler ML models like decision trees or logistic regression are preferred due to their low computational requirements. These models can be deployed on basic hardware systems and still provide timely predictions, making them suitable for emergency departments or rural clinics.

In conclusion, AI has demonstrated significant potential in the early detection and prediction of sepsis. By utilizing advanced ML algorithms and integrating diverse data sources, AI models can substantially improve the accuracy and timeliness of sepsis diagnosis. As these technologies evolve, their clinical implementation could lead to earlier interventions, better patient outcomes, and lower healthcare costs.

## 3 Methodology

This review synthesizes studies exploring the application of artificial intelligence (AI) in sepsis management, focusing on early detection, prediction, and monitoring. A structured approach was followed to ensure the comprehensive inclusion of relevant literature.

### 3.1 Search strategy

The review employed a systematic search of databases including PubMed, Scopus, and IEEE Xplore. The search terms encompassed combinations of “sepsis, ““machine learning,” “artificial intelligence,” “predictive analytics,” and “clinical decision support systems.” Filters were applied to limit results to peer-reviewed articles published in English within the last decade. Additionally, forward and backward citation tracking was performed on the included studies to ensure a thorough literature review.

### 3.2 Study selection

Titles, abstracts, and keywords were screened for relevance using criteria focused on the utilization of AI techniques for sepsis management. Full-text articles were then evaluated to confirm their alignment with the scope of the review. Studies were included if they (1) implemented AI methodologies such as machine learning or deep learning, (2) targeted sepsis-related outcomes (including early detection, prediction, or monitoring), and (3) provided detailed methodological insights or clinical applications.

### 3.3 Data extraction

Key data points extracted from each study included the type of AI models utilized, data sources, sample sizes, evaluation metrics, and reported outcomes. Information on model performance, limitations, and clinical applicability was systematically recorded to enable comparative analysis. The review prioritized studies that demonstrated real-world clinical implementations or addressed critical challenges in AI-based sepsis management.

### 3.4 Analysis framework

The included studies were categorized based on the type of AI techniques used (e.g., machine learning, natural language processing, deep learning) and their specific applications in sepsis management. The strengths and limitations of each approach were assessed to provide a nuanced understanding of the current landscape and identify areas for future research.

## 4 Diagnostic assistance through AI

AI has become a transformative tool in sepsis diagnosis, offering clinicians improved accuracy and efficiency. Traditional diagnostic methods often fail to identify sepsis promptly due to its heterogeneous presentation, resulting in delayed treatment and higher mortality.AI, utilizing ML and data analytics, significantly improves diagnostic accuracy by analyzing large volumes of clinical data to identify sepsis-indicative patterns. Beyond early detection, AI enhances clinical decision-making. By integrating real-time data from various sources, AI systems provide clinicians with actionable insights, improving diagnostic precision and guiding treatment plans.

A key advantage of AI in sepsis diagnosis is its ML ability to integrate and analyze complex datasets, such as patient demographics, vital signs, laboratory results, and clinical notes. Fleuren et al. (19) conducted a systematic review and meta-analysis, highlighting the effectiveness of models in accurately predicting sepsis in 2020.The study found that AI models achieved AUROC values between 0.68 and 0.99 across different hospital settings, showing stronger diagnostic performance compared to traditional methods. This analysis highlights AI’s potential to revolutionize sepsis diagnostics by delivering real-time, data-driven insights that improve early detection and treatment initiation (19).

Additionally, AI has demonstrated promise in improving diagnostic accuracy for neonatal and pediatric sepsis, which poses unique challenges due to nonspecific symptoms in these populations. Sweeney et al. (20) validated the Sepsis MetaScore, a gene-expression-based diagnostic test in neonates in 2018.The study showed that the Sepsis MetaScore achieved an AUROC of 0.92–0.93, significantly outperforming standard laboratory tests. This gene-expression signature provided an objective measure of sepsis risk, potentially reducing unnecessary antibiotic use and improving clinical outcomes (20). Similarly, Iregbu et al. discussed how multi-omics and AI enhance diagnostic precision for neonatal sepsis, emphasizing the need for precision medicine approaches in low-and middle-income countries to address diagnostic challenges and improve sepsis management in 2022 (21).

In recent years, significant advances have been made in applying AI to sepsis prediction and treatment.ML and deep learning models have demonstrated enhanced diagnostic accuracy, improved early detection, and provided new insights into personalized treatment strategies. Table 2 provides a summary of key studies in this domain, highlighting the AI models used and their specific applications in sepsis management. The studies included in Table 2 showcase a diverse range of AI techniques, from deep learning algorithms for early detection to ML models for real-time decision support. These approaches illustrate the growing impact of AI on sepsis care, particularly in critical care settings, emergency departments, and across multiple patient populations.

**Table 2:** Latest sepsis prediction and treatment reviews.

Title	Authors	Model	Application
Machine learning for the prediction of sepsis: a systematic review and meta-analysis of diagnostic test accuracy	Fleuren et al. (19)	Machine Learning	Sepsis prediction
Decreased intestinal microbiome diversity in pediatric sepsis: a conceptual framework for intestinal dysbiosis to influence immunometabolic function	Weiss et al. (131)	Artificial Intelligence	Early prediction of sepsis
Early prediction of sepsis from clinical data: the PhysioNet/Computing in Cardiology Challenge 2019	Reyna et al. (132)	Machine Learning	Early detection of sepsis
Medical decision support using machine learning for early detection of late-onset neonatal sepsis	Mani et al. (133)	Various Machine Learning	Sepsis prediction using EHR
Multicentre validation of a sepsis prediction algorithm using only vital sign data in the emergency department, general ward and ICU	Mao et al. (134)	Machine Learning	Sepsis prediction across multiple settings
Early ICU-acquired hypernatraemia is associated with injury severity and preceded by reduced renal sodium and chloride excretion in polytrauma patients	Rugg et al. (135)	Machine Learning	Prediction using minimal EHR data
A clinically applicable approach to continuous prediction of future acute kidney injury	Tomašev et al. (117)	Machine Learning	Predicting AKI related to sepsis
Can informatics innovation help mitigate clinician burnout?	Bakken et al. (136)	Machine Learning	Detection and management of sepsis

Beyond improving diagnostic accuracy, AI can identify biomarkers and digital signatures essential for timely and accurate sepsis diagnosis. Komorowski et al. (22) reviewed the use of ML in identifying sepsis biomarkers and developing diagnostic tools. The study highlighted the potential of combining biomarkers and clinical data using ML models to improve the timeliness and accuracy of sepsis diagnosis. This approach aids early recognition and helps understand the underlying pathophysiological mechanisms of sepsis, thereby informing targeted therapeutic strategies (22).

Moreover, AI-based diagnostic tools extend beyond hospital settings and have been developed for emergency departments and prehospital care. Kijpaisalratana et al. (16) showed that ML algorithms significantly outperformed traditional screening tools like qSOFA and SIRS in early sepsis detection among ED patients in 2022.The study found that the random forest algorithm achieved an AUROC of 0.931, highlighting AI’s potential to improve diagnostic accuracy and clinical decision-making in fast-paced environments like EDs (16).

Accurate AI diagnosis establishes the foundation for developing personalized treatment plans tailored to each patient’s unique characteristics. With precise diagnostic insights, healthcare providers can better tailor interventions to align treatments with the specific needs and physiological responses of individual patients (23). This sets the stage for AI to further enhance sepsis management through personalized treatment strategies.

In conclusion, AI demonstrates significant potential to enhance sepsis diagnostics. By integrating complex datasets and utilizing advanced ML algorithms, AI can significantly improve diagnostic accuracy, enable early detection, and reduce reliance on traditional, less accurate methods. As AI evolves, its integration into clinical practice promises better patient outcomes and more efficient healthcare delivery.

## 5 AI in personalized treatment plans

AI is increasingly used to develop personalized sepsis treatment plans, offering tailored therapeutic strategies based on individual patient characteristics. Personalized treatment aims to improve clinical outcomes by considering each patient’s unique biological and clinical profile, optimizing therapeutic efficacy and minimizing adverse effects. After accurate diagnosis, AI plays a key role in personalizing treatment strategies, which is crucial given the complexity and variability of sepsis presentations.

A major advancement in personalized sepsis treatment is the integration of ML algorithms to predict patient-specific therapy responses. Chen et al. (24) developed an autophagy-related gene classifier using ML algorithms to improve early sepsis diagnosis and prognosis in 2022.The classifier, based on eight key autophagy-related genes, showed high diagnostic accuracy and was significantly associated with immune cell infiltrations and immune pathway activations. The model distinguished sepsis from other critical illnesses and predicted patient mortality more effectively than traditional clinical characteristics, facilitating personalized treatment decisions by reflecting the immune microenvironment diversity in sepsis patients (24).

Another promising AI application in personalized sepsis treatment involves using deep reinforcement learning and simulation models. Petersen et al. (11) used deep reinforcement learning to develop an adaptive personalized treatment policy for sepsis. This approach simulated the innate immune response to infection, allowing exploration of therapeutic strategies beyond current clinical practice. The adaptive treatment policy significantly reduced mortality rates in simulated patients compared to standard antibiotic therapy, showcasing AI’s potential to improve outcomes by dynamically adjusting therapies based on real-time patient data (25).

AI also plays a vital role in identifying and categorizing sepsis endotypes, guiding individualized treatment plans. Liu et al. (26) stressed the importance of phenotyping immune functions and classifying sepsis patients into specific endotypes to create personalized treatment approaches in 2023.By understanding diverse host responses to sepsis and using ML to analyze patient data, researchers can identify signaling pathways and immune phenotypes, leading to the discovery of new therapeutic targets and the repurposing of existing drugs for sepsis treatment (26). This personalized approach ensures treatments are tailored to each patient’s unique immune responses, improving therapeutic efficacy and reducing the risk of adverse effects.

Additionally, AI-driven models have been developed to predict the individual treatment effects of specific therapies in sepsis. Pirracchio et al. (27) used ML to estimate the individual treatment effect (ITE) of corticosteroids in septic shock patients in 2020. The study showed that an individualized treatment strategy based on the optimal ITE model provided a positive net benefit, outperforming traditional scoring systems like SAPS II. This model identified patients most likely to benefit from corticosteroid therapy, optimizing treatment decisions and improving clinical outcomes (27).

Reinforcement learning (RL) models are particularly suited for developing adaptive treatment strategies in sepsis management. Unlike supervised learning models, RL algorithms can simulate dynamic clinical environments and learn optimal policies by interacting with virtual patient populations. This makes them invaluable for exploring treatment scenarios that go beyond standard clinical practice, such as optimizing fluid resuscitation or antibiotic administration in real time.

For example, RL models have been used to develop personalized treatment policies that adjust based on individual patient responses, demonstrating significant reductions in simulated mortality rates. While these models are still in experimental stages, their potential for real-time decision-making in complex and rapidly evolving conditions like sepsis is unparalleled.

As personalized treatment plans are implemented, continuous real-time monitoring is essential to adapt to the patient’s evolving condition and ensure optimal outcomes. These developments underline AI’s growing capacity to transform sepsis care by integrating predictive, adaptive, and personalized approaches to treatment.

## 6 AI in monitoring and management of sepsis

AI plays a vital role in the continuous monitoring and management of sepsis, offering real-time insights and enabling timely interventions. Integrating AI with patient monitoring systems aids in the early detection of sepsis-related complications and optimizes patient outcomes through personalized management strategies.AI enhances personalized treatments and facilitates continuous patient monitoring, allowing dynamic adjustments to treatment protocols based on real-time data.

A key application of AI in sepsis management is the use of ML models to predict and monitor sepsis-associated acute kidney injury (SA-AKI). Cheungpasitporn et al. (28) highlighted the effectiveness of supervised learning models, such as XGBoost and RNN-LSTM, in predicting SA-AKI onset and subsequent mortality. These models analyze vast datasets to uncover complex patterns beyond human discernment, enabling early risk detection and personalized management of SA-AKI. The study emphasized AI’s potential to continually refine treatment strategies based on patient outcomes, while acknowledging the ethical and practical challenges, such as data privacy and algorithmic biases, that need to be addressed.

In wearable technology, Ghias et al. (29) demonstrated the feasibility of using wearable sensors with ML models to monitor and predict sepsis mortality in low-middle-income countries (LMICs). The study found that ML models trained on heart rate variability (HRV) data from wearable sensors outperformed traditional bedside monitor models. This approach reduced sepsis mortality risk in resource-limited settings and highlighted the potential of integrating automated ML prediction models with wearable technology to improve sepsis management.

AI-driven continuous monitoring systems are being developed to provide real-time predictions of late-onset sepsis (LOS) in preterm infants. Yang et al. (30) developed an AI model that uses vital signs data from patient monitors to provide hourly LOS risk predictions. The model achieved high accuracy in detecting LOS before clinical deterioration, which is crucial for timely interventions in neonatal intensive care units (NICUs). The study showed that combining interpretability with clinical alarm management could enhance the implementation of AI models in medical practice.

Another key development is the use of digital twins and predictive analytics monitoring for sepsis management. Davis et al. (31) proposed a novel illness scoring system that uses continuous predictive analytics monitoring to track patient deterioration in real-time in 2020.This system analyzes continuous bedside monitoring data to provide early warnings of increasing sepsis risk, enabling timely and safer interventions. The study emphasized the importance of continuous risk trend analysis over traditional static alerts, which are less suited for detecting rapid clinical deterioration.

AI-based automated alert systems also show promise in improving sepsis outcomes. Zhang et al. (32) conducted a meta-analysis comparing the effectiveness of automated alerts to usual care for sepsis management. The study found that ML-based prediction methods significantly reduced sepsis mortality compared to rule-based approaches. Automated alert systems, particularly in emergency departments and hospital wards, showed a larger benefit in reducing mortality, underscoring AI’s potential to enhance early intervention and sepsis management across clinical settings (33).

In conclusion, AI has proven to be a powerful tool in continuously monitoring and managing sepsis. By using advanced ML algorithms and integrating them with wearable technology and real-time monitoring systems, AI can significantly improve early detection, personalized treatment, and overall patient outcomes. As these technologies evolve, their implementation in clinical practice promises to transform sepsis management by providing timely interventions and reducing the burden of this critical condition. By addressing these challenges, the full potential of AI in revolutionizing sepsis care can be realized, improving patient outcomes and reducing mortality rates.

## 7 AI in prognosis and outcome prediction

AI has significantly advanced prognosis and outcome prediction in sepsis, enabling healthcare providers to anticipate disease progression and tailor interventions accordingly. By analyzing continuous monitoring data, AI algorithms can predict long-term outcomes, offering clinician’s critical insights into disease progression and recovery trajectories.

A key application of AI in sepsis prognosis is developing predictive models that assess the likelihood of patient survival and recovery. Liu et al. (26) highlighted the critical role of neutrophil-endothelial cell interactions in sepsis progression and used ML models to identify specific immune phenotypes linked to different outcomes. These models used data from organ-on-chip experiments, omics analyses, and clinical records to predict patient prognosis and identify therapeutic targets. Similarly, Pirracchio et al. (27) used ML to estimate the ITE of corticosteroids in septic shock patients, showing that AI-based individualized treatment strategies could provide a positive net benefit and improve survival rates in 2020.

Beyond individual treatment effects, AI models have been used to predict long-term outcomes in sepsis survivors. Wang et al. (34) discussed AI-driven clinical decision support tools to predict long-term functional outcomes in pediatric sepsis patients. These tools integrate EHRs, physiological data, and treatment histories to predict recovery and long-term complications. The study showed that AI models significantly outperformed traditional prognostic scores, providing more precise and individualized predictions. Similarly, Sweeney et al. (20) validated the Sepsis MetaScore, a gene-expression-based prognostic tool, which provided highly accurate predictions of sepsis outcomes in neonates, further showcasing AI’s utility in prognostic modeling in 2018.

AI’s ability to analyze vast amounts of data also extends to identifying sepsis sub-phenotypes and their associated outcomes. Komorowski et al. (22) reviewed ML applications in characterizing sepsis sub-phenotypes based on biomarkers and clinical data in 2022. By identifying distinct subgroups of sepsis patients, AI models can predict disease trajectories and tailor interventions to individual patient needs. This approach improves prognostic accuracy and facilitates the development of targeted therapies to enhance patient outcomes. Cheungpasitporn et al. (28) highlighted the use of unsupervised learning techniques to uncover clinically relevant sub-phenotypes in sepsis-associated acute kidney injury (SA-AKI) patients, enabling more personalized and effective management strategies.

Additionally, AI-based prognostic models are being integrated into real-time clinical workflows to support dynamic risk assessment and management. Zhang et al. (35) conducted a meta-analysis comparing automated alert systems to usual care in sepsis management. The study found that ML-based alert systems significantly reduced sepsis mortality by offering continuous risk assessments and timely interventions. This real-time integration of AI models into clinical practice enhances the ability to monitor patient status and predict adverse outcomes, improving clinical outcomes. Similarly, Davis et al. (31) proposed continuous predictive analytics monitoring, which uses AI to track patient deterioration and provide early warnings of critical events, optimizing sepsis management and reducing mortality.

Deep learning models, particularly convolutional and recurrent neural networks, are ideal for predicting the progression of sepsis and its complications, such as sepsis-associated acute kidney injury (SA-AKI) or acute respiratory distress syndrome (ARDS). These models excel at processing complex, high-dimensional data, such as time-series data from continuous monitoring systems or unstructured clinical notes. For example, recurrent neural networks (RNNs) are particularly effective in ICU settings where temporal patterns in vital signs or laboratory results are critical for predicting disease trajectories (12).

Deep learning’s ability to integrate multimodal data—combining EHRs, imaging, and genomic data—further enhances its applicability for personalized sepsis care. However, their “black-box” nature requires additional tools, such as attention mechanisms, to improve interpretability for clinical use (36).

In conclusion, AI has transformed sepsis prognosis and outcome prediction by providing accurate, individualized, real-time insights into disease progression and patient outcomes. By integrating advanced ML algorithms with comprehensive patient data, AI models can significantly enhance prognostic accuracy, guide personalized treatments, and improve overall clinical outcomes in sepsis management. By addressing these challenges, the full potential of AI to revolutionize sepsis care can be realized, leading to improved patient outcomes and reduced mortality rates.

## 8 Results and discussion

Artificial Intelligence (AI) has emerged as a transformative tool in sepsis management, offering promising advancements in early detection, outcome prediction, and personalized treatment. Machine learning (ML) and deep learning models have demonstrated significant improvements over traditional methods by analyzing complex datasets and identifying patterns often missed by conventional diagnostic tools. For example, AI algorithms, such as random forests and deep neural networks, have shown the ability to predict sepsis onset hours before clinical symptoms appear, with some models achieving AUROC values above 0.90. These capabilities enable timely interventions, which are critical in reducing sepsis-related mortality and improving patient outcomes. Furthermore, advanced predictive models have been developed to forecast disease progression and complications, such as sepsis-associated acute kidney injury (SA-AKI) and acute respiratory distress syndrome (ARDS), thereby supporting informed clinical decision-making (36).

AI’s transformative potential in sepsis management is further underscored by its ability to integrate diverse data sources for clinical purposes such as early warning, treatment optimization, and monitoring. Emerging data types include transcriptomic profiles, proteomics, imaging data, and unstructured text from electronic health records (EHRs). These rich datasets enable AI models to identify sepsis subgroups, predict treatment responses, and tailor therapeutic strategies. For instance, Zhang et al. (32) utilized multi-omics integration to identify septic shock subgroups, leveraging transcriptomic and proteomic data to guide fluid management strategies, achieving a predictive accuracy with an AUC of 0.802. Additionally, Wang et al. (7) demonstrated the importance of fluid balance trajectories derived from longitudinal ICU data, revealing their strong association with hospital mortality and organ dysfunction.

These examples highlight the increasing utility of advanced datasets in enhancing the precision of AI models for sepsis care. Transcriptomic data provide insights into cellular responses and systemic inflammation, while imaging data can reveal structural changes indicative of complications. Unstructured clinical notes contribute valuable contextual information, enabling comprehensive decision support. By combining these data modalities, AI has the potential to transform sepsis management from reactive to predictive and personalized approaches.

Despite these advancements, challenges persist in the implementation of AI for sepsis management. A major limitation is the lack of generalizability across diverse clinical settings. AI models trained on datasets from specific populations often exhibit decreased accuracy when applied to new or varied demographic and geographic contexts. This variability underscores the need for more diverse and inclusive datasets to enhance the robustness and adaptability of AI tools. Additionally, many AI models rely on static data, limiting their utility in dynamic clinical environments where patient conditions can change rapidly. Developing real-time adaptive AI systems capable of continuous learning and updating based on new data is critical for achieving broader clinical applicability.

The discussion would be incomplete without addressing the comparison between AI and traditional diagnostic tools for sepsis. While tools like the Sequential Organ Failure Assessment (SOFA) and the Modified Early Warning Score (MEWS) remain integral to clinical workflows, AI has consistently outperformed these methods in terms of sensitivity, specificity, and efficiency. For instance, AI-based systems can analyze vast amounts of electronic health record (EHR) data in real time, enabling faster and more accurate diagnoses compared to traditional rule-based approaches. However, it is important to acknowledge that traditional tools provide a foundation for validating AI systems and serve as benchmarks for measuring AI’s performance in clinical practice.

Ethical and regulatory considerations are also central to the adoption of AI in sepsis care. Data privacy and algorithmic transparency are critical concerns, especially given the sensitivity of medical data and the complexity of AI decision-making processes. Regulatory frameworks must strike a balance between encouraging innovation and ensuring patient safety. Additionally, algorithmic biases, often resulting from imbalanced training datasets, pose a risk of exacerbating disparities in healthcare outcomes. Addressing these biases through continuous monitoring, validation, and inclusive model development is essential to ensure equitable access and effectiveness of AI tools in diverse populations.

In summary, while AI has shown immense potential in transforming sepsis care, critical challenges related to generalizability, adaptability, and ethical considerations remain. By addressing these limitations and integrating AI with existing diagnostic tools, healthcare providers can harness its full potential to revolutionize sepsis management, ultimately improving patient outcomes and reducing the global burden of this life-threatening condition.

The suitability of different AI models varies significantly depending on the clinical context, with each offering distinct advantages tailored to specific settings. In intensive care units (ICUs), advanced models such as deep learning and reinforcement learning excel due to the availability of data-rich environments. These settings often feature continuous monitoring systems, allowing these sophisticated models to provide precise predictions and dynamically adjust treatment strategies in real time. Conversely, in emergency departments or resource-limited settings, simpler machine learning models are more appropriate. These models are valued for their computational efficiency and ease of implementation, enabling rapid deployment and effective decision support even in environments with constrained resources or limited access to advanced infrastructure. This flexibility across contexts underscores the importance of selecting AI models that align with the specific demands and capabilities of different clinical settings.

Artificial Intelligence (AI) has emerged as a transformative tool in sepsis management, offering promising advancements in early detection, outcome prediction, and personalized treatment. Machine learning (ML) and deep learning models have demonstrated significant improvements over traditional methods by analyzing complex datasets and identifying patterns often missed by conventional diagnostic tools (37, 38). For example, AI algorithms, such as random forests and deep neural networks, have shown the ability to predict sepsis onset hours before clinical symptoms appear, with some models achieving AUROC values above 0.90 (39, 40). These capabilities enable timely interventions, which are critical in reducing sepsis-related mortality and improving patient outcomes (41). Furthermore, advanced predictive models have been developed to forecast disease progression and complications, such as sepsis-associated acute kidney injury (SA-AKI) and acute respiratory distress syndrome (ARDS), thereby supporting informed clinical decision-making (42, 43).

AI’s transformative potential in sepsis management is further underscored by its ability to integrate diverse data sources for clinical purposes such as early warning, treatment optimization, and monitoring. Emerging data types include transcriptomic profiles, proteomics, imaging data, and unstructured text from electronic health records (EHRs) (44, 45). These rich datasets enable AI models to identify sepsis subgroups, predict treatment responses, and tailor therapeutic strategies. For instance, Yang et al. utilized multi-omics integration to identify septic shock subgroups, leveraging transcriptomic and proteomic data to guide fluid management strategies, achieving a predictive accuracy with an AUC of 0.802 (46). Additionally, Bravi et al. demonstrated the importance of fluid balance trajectories derived from longitudinal ICU data, revealing their strong association with hospital mortality and organ dysfunction (47).

These examples highlight the increasing utility of advanced datasets in enhancing the precision of AI models for sepsis care. Transcriptomic data provide insights into cellular responses and systemic inflammation, while imaging data can reveal structural changes indicative of complications (48, 49). Unstructured clinical notes contribute valuable contextual information, enabling comprehensive decision support (50). By combining these data modalities, AI has the potential to transform sepsis management from reactive to predictive and personalized approaches (51, 52).

Despite these advancements, challenges persist in the implementation of AI for sepsis management. A major limitation is the lack of generalizability across diverse clinical settings. AI models trained on datasets from specific populations often exhibit decreased accuracy when applied to new or varied demographic and geographic contexts (53, 54). This variability underscores the need for more diverse and inclusive datasets to enhance the robustness and adaptability of AI tools (55). Additionally, many AI models rely on static data, limiting their utility in dynamic clinical environments where patient conditions can change rapidly (17). Developing real-time adaptive AI systems capable of continuous learning and updating based on new data is critical for achieving broader clinical applicability (56).

The discussion would be incomplete without addressing the comparison between AI and traditional diagnostic tools for sepsis. While tools like the Sequential Organ Failure Assessment (SOFA) and the Modified Early Warning Score (MEWS) remain integral to clinical workflows, AI has consistently outperformed these methods in terms of sensitivity, specificity, and efficiency (57, 58). For instance, AI-based systems can analyze vast amounts of electronic health record (EHR) data in real time, enabling faster and more accurate diagnoses compared to traditional rule-based approaches (59, 60). However, it is important to acknowledge that traditional tools provide a foundation for validating AI systems and serve as benchmarks for measuring AI’s performance in clinical practice (61).

Ethical and regulatory considerations are also central to the adoption of AI in sepsis care. Data privacy and algorithmic transparency are critical concerns, especially given the sensitivity of medical data and the complexity of AI decision-making processes (33, 62). Regulatory frameworks must strike a balance between encouraging innovation and ensuring patient safety (63). Additionally, algorithmic biases, often resulting from imbalanced training datasets, pose a risk of exacerbating disparities in healthcare outcomes. Addressing these biases through continuous monitoring, validation, and inclusive model development is essential to ensure equitable access and effectiveness of AI tools in diverse populations (64).

In summary, while AI has shown immense potential in transforming sepsis care, critical challenges related to generalizability, adaptability, and ethical considerations remain. By addressing these limitations and integrating AI with existing diagnostic tools, healthcare providers can harness its full potential to revolutionize sepsis management, ultimately improving patient outcomes and reducing the global burden of this life-threatening condition (65, 66).

The suitability of different AI models varies significantly depending on the clinical context, with each offering distinct advantages tailored to specific settings. In intensive care units (ICUs), advanced models such as deep learning and reinforcement learning excel due to the availability of data-rich environments (67, 68). These settings often feature continuous monitoring systems, allowing these sophisticated models to provide precise predictions and dynamically adjust treatment strategies in real time (69). Conversely, in emergency departments or resource-limited settings, simpler machine learning models are more appropriate. These models are valued for their computational efficiency and ease of implementation, enabling rapid deployment and effective decision support even in environments with constrained resources or limited access to advanced infrastructure (46, 70). This flexibility across contexts underscores the importance of selecting AI models that align with the specific demands and capabilities of different clinical settings (71).

## 9 Ethical and practical considerations

The integration of Artificial Intelligence (AI) into sepsis management presents significant ethical and practical challenges that must be carefully addressed to ensure its sustainable adoption in clinical practice. While AI holds transformative potential, considerations around data privacy, algorithmic bias, and model transparency are critical to building trust and ensuring equitable healthcare delivery.

### 9.1 Data privacy and governance

AI algorithms rely on vast amounts of patient data, raising concerns about privacy and data security (72, 73). Sensitive information from electronic health records (EHRs), genomic datasets, and real-time monitoring systems must be safeguarded to prevent breaches and unauthorized access (74). Establishing robust data governance frameworks is essential to address these risks. Frameworks such as the Health Insurance Portability and Accountability Act (HIPAA) in the United States and the General Data Protection Regulation (GDPR) in Europe provide clear guidelines for handling patient data responsibly (75, 76). Future initiatives should also emphasize the anonymization of datasets and the use of secure, decentralized storage systems to enhance data protection while enabling large-scale AI model training (77).

### 9.2 Algorithmic bias and equity

Algorithmic bias poses a significant challenge in ensuring fair and equitable healthcare outcomes (78, 79). AI models trained on biased datasets—such as those over-representing specific demographics or healthcare settings—may perform poorly in underrepresented populations (80). For example, sepsis prediction models developed primarily using data from high-resource hospitals may fail to generalize to low-resource settings. To mitigate this, developers should prioritize diverse and representative datasets during training and validation phases (81, 82). Continuous monitoring and auditing of AI models are also necessary to identify and correct biases, ensuring they perform equitably across different populations (83).

### 9.3 Transparency and explainability

The “black-box” nature of many AI models, particularly deep learning algorithms, creates barriers to clinician trust and adoption (84, 85). Clinicians often require interpretable insights to make informed decisions, particularly in high-stakes scenarios like sepsis management (86). Explainable AI (XAI) models address this by providing human-readable explanations of predictions (87, 88). For instance, attention mechanisms in deep learning models can highlight the most relevant features influencing a diagnosis, while rule-based systems can offer clear decision pathways (89). Encouraging the use of XAI will not only enhance clinician trust but also facilitate regulatory approval by demonstrating accountability and fairness (90).

### 9.4 Practical strategies for clinical integration

Practical integration of AI systems into clinical workflows requires careful consideration of usability and interoperability (91, 92). AI tools must be seamlessly integrated with existing healthcare infrastructure, such as EHR systems, to minimize disruptions (93). User-friendly interfaces are critical for ensuring that clinicians can easily interpret and act on AI-generated insights. Additionally, training programs tailored for healthcare providers are essential to enhance their understanding of AI capabilities and limitations (94, 95). Collaborative efforts between AI developers, clinicians, and regulatory bodies will be key to addressing these challenges and fostering acceptance.

### 9.5 Balancing innovation with ethical responsibility

While AI offers immense potential to revolutionize sepsis care, it is imperative to balance innovation with ethical responsibility. Regulatory frameworks should evolve to include AI-specific guidelines that emphasize safety, efficacy, and fairness (96, 97). Interdisciplinary collaboration between data scientists, ethicists, clinicians, and policymakers will ensure that AI systems align with clinical needs and ethical standards. Such efforts will pave the way for sustainable and responsible AI integration into sepsis management (98, 99).

By addressing data privacy, algorithmic bias, model transparency, and practical integration challenges, AI can be responsibly and equitably adopted to enhance sepsis care, fostering innovation while ensuring patient safety and trust.

## 10 Limitations of applying artificial intelligence

### 10.1 Limited universal applicability

**Figure 2:**
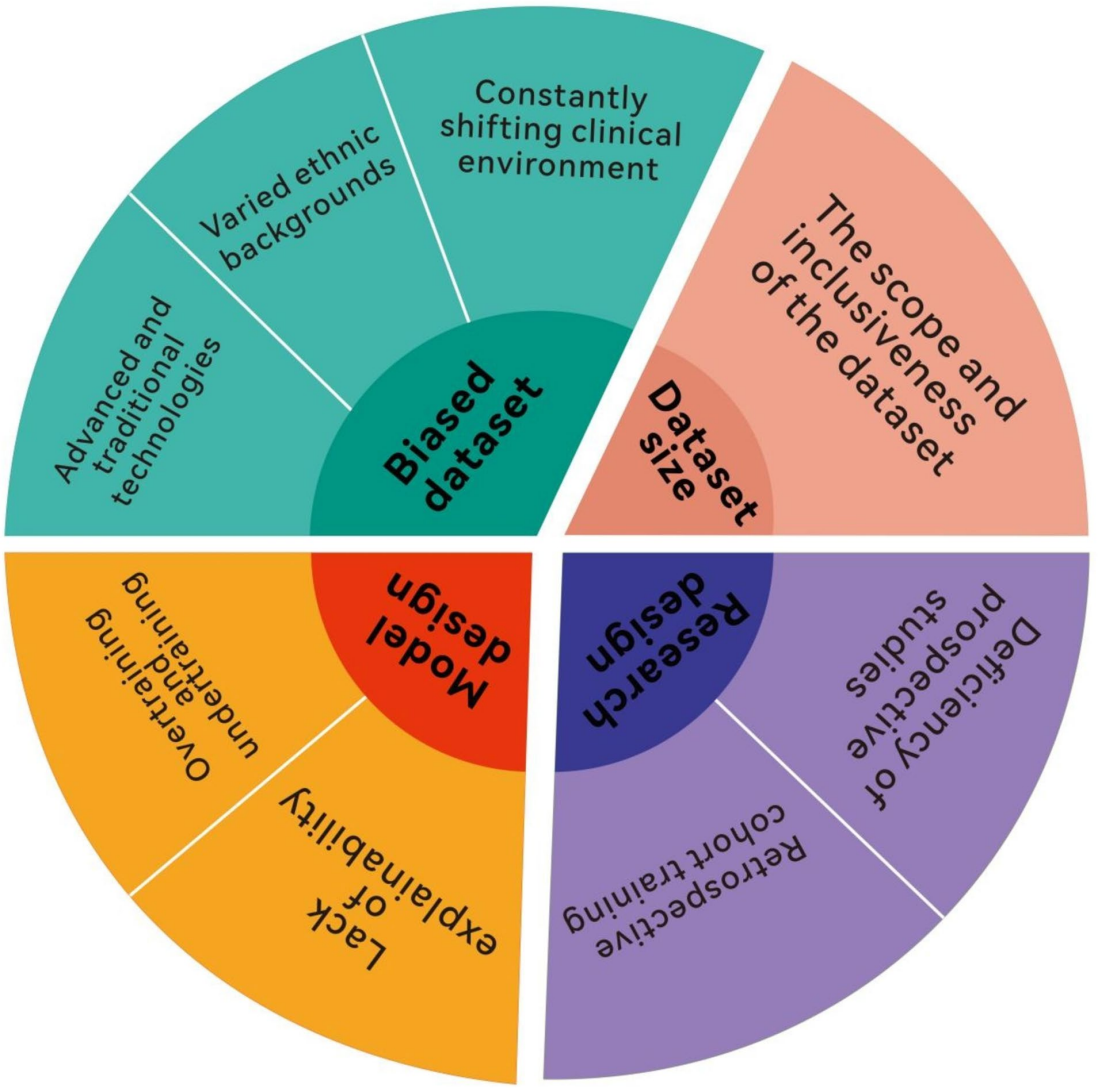
Limitations of applying artificial intelligence.

Despite AI’s transformative potential in leveraging clinical data and its critical role in diagnosis and treatment, substantial challenges remain (Figure 2). One pressing obstacle is ensuring AI models are universally applicable across diverse clinical environments. The limited universal applicability of AI models is a critical issue. When a model developed in one hospital is applied in another, the expected outcomes often fail due to differences in workflows, software, hardware, database structures, patient demographics, and disease epidemiology across institutions (100, 101). For example, variations in care practices, technology infrastructure, and population health further complicate replicating AI success between facilities (40, 102). At the University of Michigan Hospital, the AI system experienced alert overload as patient demographics shifted during the COVID-19 pandemic, leading to increased clinical burden and suspension of the model in 2020 (41, 42, 103, 104). This incident highlights broader concerns about how unforeseen changes in patient characteristics or institutional practices can overwhelm advanced AI systems. This underscores that building AI models is not static; continual adaptation to patient populations, disease progression, clinical guidelines, and hospital environments is essential (43, 105). While these models demonstrate significant potential, their effective application requires careful matching of model strengths to clinical needs. Future research should focus on integrating these models into hybrid frameworks that combine their strengths, such as using ML for initial detection and RL for adaptive treatment. Moreover, real-world validation in diverse settings is essential to ensure generalizability and scalability. To ensure lasting clinical utility, AI models may require continuous self-updates, enabling them to evolve with changing clinical landscapes, enhancing universal applicability and bridging the gap between “computational” and “clinical medicine” (45, 106).

### 10.2 Limited clinical applicability

Another key issue is the perceived limited clinical relevance of AI models among medical professionals. Despite technological advancements, AI adoption in healthcare is met with skepticism, mainly due to the mismatch between complex algorithms and practical clinical needs. Studies show healthcare personnel often lack a full understanding of AI due to its complex logic, which may not align with traditional medical reasoning (44, 107). This disconnect can hinder the seamless integration of AI tools into daily medical practice, particularly in critical care settings. In predictive models, for instance, alerts triggered before illness onset may not inspire confidence among clinicians, as patients have not yet shown clinical deterioration (46, 108). Such early alerts may seem unnecessary or premature, reducing trust and engagement from healthcare teams. Consequently, only 12% of doctors and 38% of nurses believe AI models improve diagnostic and treatment services (47, 109). However, recent research suggests that if physicians review and confirm sepsis alerts within 3 h, antibiotic administration time can be reduced by an average of 1.8 h. This shows that timely validation of AI alerts by clinicians can significantly improve treatment efficiency. Thus, beyond improving AI model accuracy, attention must focus on medical staff’s attitudes toward AI (19). Resistance or hesitation from clinicians can pose a significant barrier to AI integration into routine clinical workflows. Healthcare professionals’ awareness, experience, and acceptance of AI are crucial to its practical effectiveness (49, 110). Future research should strengthen trust in AI models, align them with clinical diagnostic processes, and thoroughly evaluate their real impact on clinical outcomes (50, 111). Greater transparency in how AI models reach conclusions may also help bridge the trust gap between technology and medical professionals.

In short, enhancing the clinical relevance and acceptance of AI in sepsis management requires addressing the trust gap, aligning AI functionalities with practical clinical needs, and fostering collaboration between medical professionals and AI systems to improve patient outcomes.

## 11 Future directions and research opportunities

In summary, AI offers transformative potential across the sepsis care continuum, from early detection to real-time monitoring and outcome prediction. By addressing current challenges and leveraging AI capabilities, we can significantly improve patient outcomes and revolutionize sepsis management. The integration of AI technologies in sepsis care has shown promising results in improving early detection, facilitating personalized treatment, and predicting patient outcomes. Ongoing efforts are needed to overcome challenges related to ethics, clinical integration, and algorithmic bias to fully realize AI’s potential in sepsis care.

One promising direction is enhancing AI algorithms by incorporating more diverse and comprehensive datasets. Current AI models often rely on data from specific populations or healthcare settings, limiting their generalizability. Future research should focus on integrating data from diverse patient populations, including those from different regions, socioeconomic backgrounds, and comorbidities. This will help develop more robust and universally applicable AI models. Liu et al. (26) highlighted the importance of diverse datasets to reduce algorithmic biases and improve AI prediction accuracy across different patient groups. Collaborative efforts between global healthcare institutions can facilitate the creation of large, diverse datasets for training and validating AI models (26).

Another critical research area is developing real-time, adaptive AI systems. Current AI models provide static predictions based on historical data, but sepsis’s dynamic nature requires continuous monitoring and real-time decision support. Future AI systems should continuously learn and adapt to new data, providing up-to-the-minute insights and recommendations. Davis et al. (31) discussed the potential of continuous predictive analytics monitoring, which uses real-time data to predict patient deterioration and guide timely interventions. These systems could significantly improve sepsis management by enabling healthcare providers to respond swiftly to changes in patient conditions (31).

Integrating AI with emerging technologies like digital twins and wearable devices presents another exciting research opportunity. Digital twins, virtual replicas of physical systems, can simulate and predict sepsis progression and treatment outcomes in individual patients. Dang et al. (112) described using digital twin models in neurocritical care, suggesting that similar approaches could apply to sepsis management. Combining digital twins with AI allows clinicians to test various treatment scenarios and optimize strategies for each patient. Additionally, wearable devices with sensors can continuously monitor vital signs and other health indicators, feeding data into AI systems for real-time analysis and early sepsis detection (113).

Ethical and regulatory considerations present ongoing research opportunities. As AI becomes more integrated into clinical practice, addressing data privacy, security, and ethical use of AI technologies is crucial. He et al. (36). emphasized the need for robust data governance frameworks and clear ethical guidelines to protect patient privacy while enabling effective AI use. Research should also focus on developing transparent and interpretable AI models clinicians can trust and understand, ensuring AI-driven decisions are made with accountability and transparency (36).

Finally, interdisciplinary collaboration is essential to advancing AI in sepsis management. Researchers, clinicians, data scientists, and regulators must collaborate to address challenges and leverage AI opportunities. Collaborative research initiatives and shared resources can accelerate AI development and implementation, ultimately leading to improved patient outcomes. Pirracchio et al. (27). highlighted the importance of stakeholder collaboration in creating regulatory frameworks that balance innovation with patient safety, ensuring AI tools are effective and compliant with ethical standards (27).

In conclusion, the future of AI in sepsis management holds great promise, with many research opportunities to enhance its capabilities and applications. By focusing on diverse datasets, real-time adaptive systems, emerging technologies, ethical considerations, and interdisciplinary collaboration, AI’s potential to transform sepsis care can be fully realized. These efforts will pave the way for more accurate, personalized, and effective sepsis management, ultimately improving patient outcomes and saving lives.

## 12 Conclusion

The integration of Artificial Intelligence (AI) in sepsis management demonstrates significant promise in transforming diagnosis, treatment, and overall outcomes for this life-threatening condition. By leveraging advanced machine learning (ML) algorithms and large datasets, AI enhances early detection, provides diagnostic assistance, personalizes treatment plans, monitors patient status in real time, and predicts outcomes with high accuracy. This section explores AI’s role across these dimensions, highlighting advancements, challenges, and future directions.

AI-driven early detection and prediction models have shown the ability to identify sepsis at its onset, enabling timely interventions crucial for reducing mortality. Studies by Wang et al. (7) and Bai et al. (12) demonstrate that AI models achieve high accuracy in predicting sepsis in ICU patients by analyzing clinical variables from electronic health records (EHRs). These advancements underscore AI’s potential to significantly improve early sepsis detection, enhancing patient outcomes through timely and effective treatment. Building on the capabilities of early detection, AI also supports diagnostic assistance by enhancing accuracy and efficiency in identifying sepsis. Fleuren et al. (19) highlighted the efficacy of ML models in predicting sepsis with high accuracy across different hospital settings. Additionally, tools like the Sepsis MetaScore validated by Sweeney et al. (20) provide objective measures of sepsis risk, reducing unnecessary antibiotic use and improving clinical outcomes in neonatal sepsis. These examples illustrate how AI assists clinicians in making more informed and precise diagnostic decisions.

Personalized treatment is another transformative application of AI in sepsis care. By analyzing individual patient data, AI models predict responses to specific therapies and tailor treatment strategies accordingly. Chen et al. (24) developed an autophagy-related gene classifier that demonstrated high diagnostic accuracy and predicted patient mortality, facilitating personalized treatment decisions. Similarly, Petersen et al. (11) used deep reinforcement learning to develop an adaptive personalized treatment policy for sepsis, significantly reducing mortality rates in simulated patients. Personalized treatment gains further depth through immune cell profiling, which offers insights into the dysregulated immune responses characteristic of sepsis. Immune cell subtypes, such as CD4+ T cells and regulatory T cells (Tregs), play critical roles in this response and serve as biomarkers for guiding precision treatment (114–116). For instance, Liu et al. classified sepsis patients based on neutrophil-endothelial cell interactions and immune signaling pathways, enabling the identification of distinct endotypes linked to clinical outcomes (26). These advancements emphasize AI’s potential to optimize therapeutic efficacy and improve patient outcomes through tailored treatment approaches.

Real-time monitoring and management represent another critical dimension of AI’s role in sepsis care. AI-driven systems such as wearable sensors and ML models proposed by Ghiasi et al. (29) have shown the feasibility of monitoring and predicting sepsis mortality in low-resource settings. Continuous predictive analytics, as demonstrated by Davis et al. (31), provide early warnings of patient deterioration, enabling timely interventions and optimizing management strategies. These innovations underscore the importance of real-time data analysis in improving sepsis care, particularly in rapidly evolving clinical scenarios.

Despite these advancements, significant challenges remain in implementing AI for sepsis management. A major limitation is the lack of generalizability across diverse clinical settings, as models trained on specific datasets often struggle to perform accurately in varied populations. Additionally, many AI systems rely on static data, limiting their utility in dynamic clinical environments where patient conditions evolve rapidly (11, 117). Addressing these issues requires more diverse and inclusive datasets, real-time adaptive systems capable of continuous learning, and collaborative efforts between healthcare institutions to create comprehensive training datasets (118).

Future research must also focus on integrating AI with emerging technologies such as digital twins, wearable devices, and multi-omics data to enhance predictive and personalized sepsis care. Ethical and regulatory considerations, including algorithmic transparency and data privacy, must be addressed to ensure sustainable and equitable adoption of AI tools in clinical practice. By overcoming these challenges, AI can fully realize its transformative potential in sepsis management.

In conclusion, AI offers a multifaceted approach to revolutionizing sepsis care. From enhancing early detection to providing diagnostic assistance, personalizing treatment plans, enabling real-time monitoring, and predicting patient outcomes, AI continues to drive significant advancements. By addressing current limitations and leveraging these opportunities, healthcare providers can harness AI’s potential to improve sepsis outcomes, ultimately saving lives and transforming sepsis management on a global scale.
